# Viral fitness cost prevents HIV-1 from evading dolutegravir drug pressure

**DOI:** 10.1186/1742-4690-10-22

**Published:** 2013-02-22

**Authors:** Thibault Mesplède, Peter K Quashie, Nathan Osman, Yingshan Han, Diane N Singhroy, Yolanda Lie, Christos J Petropoulos, Wei Huang, Mark A Wainberg

**Affiliations:** 1 McGill University AIDS Centre, Lady Davis Institute for Medical Research, Jewish General Hospital, Montreal, Quebec, Canada; 2 Division of Experimental Medicine, Faculty of Medicine, McGill University, Montreal, Quebec, Canada; 3 Department of Microbiology and Immunology, Faculty of Medicine, McGill University, Montreal, Quebec, Canada; 4 Monogram Biosciences, South San Francisco, California, USA

**Keywords:** HIV integrase, Dolutegravir, Resistance to antiretrovirals, Viral fitness, Strand-transfer assay

## Abstract

**Background:**

Clinical studies have shown that integrase strand transfer inhibitors can be used to treat HIV-1 infection. Although the first-generation integrase inhibitors are susceptible to the emergence of resistance mutations that impair their efficacy in therapy, such resistance has not been identified to date in drug-naïve patients who have been treated with the second-generation inhibitor dolutegravir. During previous *in vitro* selection study, we identified a R263K mutation as the most common substitution to arise in the presence of dolutegravir with H51Y arising as a secondary mutation. Additional experiments reported here provide a plausible explanation for the absence of reported dolutegravir resistance among integrase inhibitor-naïve patients to date.

**Results:**

We now show that H51Y in combination with R263K increases resistance to dolutegravir but is accompanied by dramatic decreases in both enzymatic activity and viral replication.

**Conclusions:**

Since H51Y and R263K may define a unique resistance pathway to dolutegravir, our results are consistent with the absence of resistance mutations in antiretroviral drug-naive patients treated with this drug.

## Background

Although highly active antiretroviral therapy (HAART) is lifesaving for people infected by the human immunodeficiency virus (HIV), the long-term efficacy of HAART is limited by drug resistance [1]. This problem exists in both resource-limited [2] and developed countries [3]. The addition of integrase strand transfer inhibitors (INSTIs) to the arsenal of antiretroviral drugs represents an important advance for the treatment of HIV-positive patients [4-9]. Raltegravir (RAL) and elvitegravir (EVG) are the first INSTIs approved for therapy [10,11] while dolutegravir (DTG) is in advanced phase 3 clinical trials [12]. Although both RAL and EVG, the first INSTIs, are susceptible to virological failure due to the emergence of resistance mutations within the integrase coding sequence [6,13,14], major resistance mutations have not been reported in drug-naive patients who were treated in clinical trials with the second-generation drug, DTG [6,12,15,16]. However, in vitro drug selection experiments performed in our laboratory have identified a R263K mutation within the integrase coding region as a resistance mutation when subtype B viruses were cultivated in the presence of DTG and also showed that H51Y commonly emerged as a secondary mutation [17]. Both of these mutations have also been selected *in vitro* with EVG and metabolites of EVG although neither is considered to be an important mutation for the latter drug [18-20]. In addition, H51Y was detected in highly treatment-experienced patients failing EVG-containing regimens [21].

The current work was carried out to further characterize resistance against INSTIs and especially DTG. A common pattern of resistance involving INSTIs and members of other drug classes, including some protease inhibitors (PIs) and nucleoside reverse transcriptase inhibitors (NRTIs), is that a first mutation imparts a minimal level of drug resistance that is accompanied by a loss of enzymatic activity, as well as a diminution in viral replication capacity. We show here that the H51Y mutation in combination with R263K increased resistance to DTG, over that conferred by R263K alone, and was accompanied by a dramatic decrease in integrase strand transfer enzymatic activity, viral replicative fitness, and the ability of HIV DNA to integrate into host cell genomes. In contrast, H51Y on its own did not affect any of these various activities. In view of the possibility that H51Y and R263K may define a unique resistance pathway against DTG, our results provide an explanation for the absence of drug resistance mutations in drug-naive patients who have been treated with DTG.

## Results

### The addition of H51Y to R263K increases resistance against dolutegravir

We have previously shown and confirm here that the unique R263K mutation confers low-level resistance (≈10-fold) to DTG (Table 1) [17]. Now, by introducing the H51Y mutation alone or in combination with R263K into pNL4.3 proviral DNA, we show that the combination of H51Y and R263K increased resistance to DTG (FC=16.5-fold), whereas H51Y alone did not confer resistance to this drug (Table 1). Similar experiments with RAL showed that the combination of both mutations conferred low-level resistance to this drug (2.1-fold, Table 1) while the individual H51Y and R263K mutations were innocuous. Notably, the fold change in RAL susceptibility observed with H51Y (1.2-fold) was not significant in our experiments but was identical to results from another study [20]. As expected, HIV susceptibility to the non-nucleoside reverse transcriptase inhibitor efavirenz (EFV) was unaltered by these mutations alone or in combination.

**Table 1:** **Effects of the H51Y and R263K mutations on IC**_**50**_**s and 95% confidence intervals for dolutegravir (DTG), raltegravir (RAL), and efavirenz (EFV)**

		**DTG**			**RAL**			**EFV**		
			95%			95%			95%	
		IC_50_	confidence		IC_50_	confidence		IC_50_	confidence	
Backbone	Genotype	(nM)	intervals(nM)	FC	(nM)	intervals(nM)	FC	(nM)	intervals(nM)	FC
pNL4-3	WT	6.897	5.714 to 8.324	-	11.51	9.833 to 13.46	-	1.352	0.964 to 1.897	-
	H51Y	9.278	7.707 to 11.17	1.3	13.42	11.62 to 15.50	1.2	1.373	0.957 to 1.970	1
	R263K	73.36	29.91 to 180	10.6	12.44	10.06 to 15.38	1.1	1.143	0.763 to 1.712	0.8
	H51Y/R263K	113.8	77.43 to 167.2	16.5	24.28	15.51 to 38.00	2.1	1.212	0.852 to 1.723	0.9

Since levels of drug resistance can vary depending on differences in assays or target cells, HIV susceptibility to RAL, EVG, and DTG was also tested in viruses containing the H51Y and R263K mutations using the PhenoSense® Integrase phenotypic assay (Monogram Biosciences) (Table 2). In this assay, R263K conferred low-level resistance to DTG (~2-fold) and the addition of H51Y to R263K further increased HIV resistance to ~7-fold. These mutations tested individually had no impact on HIV susceptibility to RAL whereas the H51Y/R263K combination conferred an approximate 3-fold resistance to this compound. Both H51Y and R263K individually conferred low-level resistance to EVG (2.06 and 3.28-fold, respectively) while the combination dramatically increased the IC_50_ for this drug by 41.5-fold.

**Table 2:** Effects of the H51Y and R263K mutations on HIV replication capacity and susceptibility to dolutegravir (DTG), raltegravir (RAL), and elvitegravir (EVG) as measured by the PhenoSense® Integrase assay (Monogram Biosciences)

		**DTG**	**RAL**	**EVG**	**Replication capacity**
Backbone	Genotype	Fold change	Fold change	Fold change	
pNL4-3	WT	0.92	0.91	1.03	100%
	H51Y	1.25	1.11	2.06	89%
	R263K	1.95	1.21	3.28	70%
	H51Y/R263K	6.95	2.94	41.5	11%

### The addition of H51Y to R263K decreases integrase strand-transfer activity

**Figure 1:**
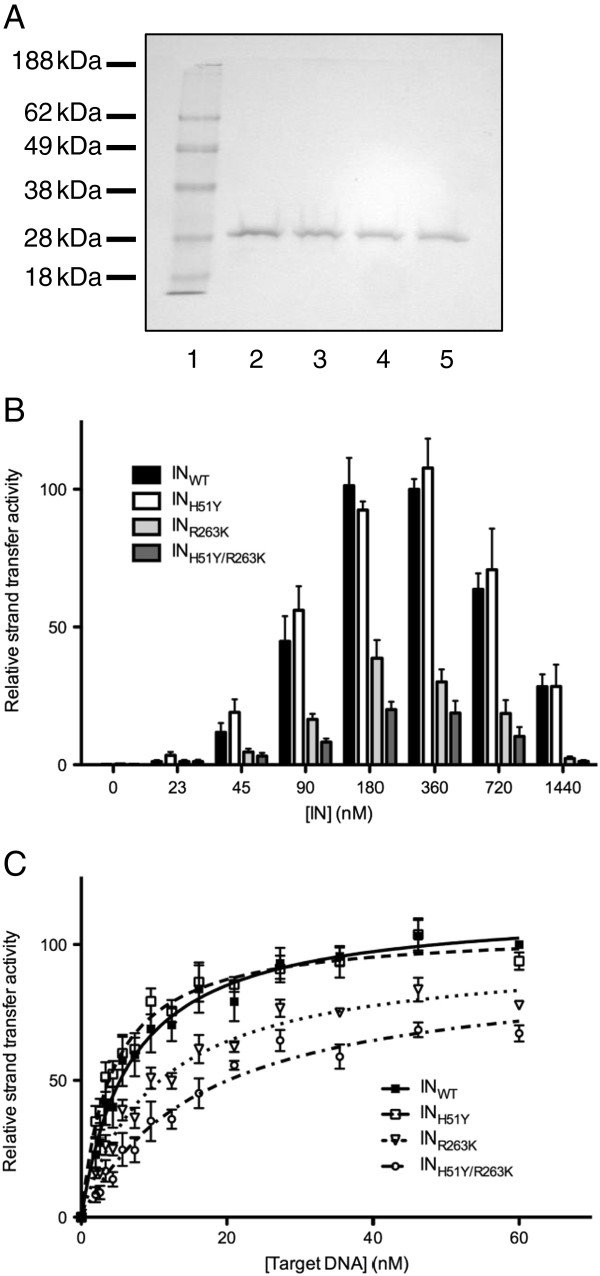
**Strand-transfer activities of purified recombinant integrase proteins.** (**A**) Recombinant integrase proteins IN_WT_, IN_H51Y_, IN_R263K_, and IN_H51Y/R263K_ were purified (lanes 2 to 5) and (**B**) used to measure strand-transfer activity in relative fluorescent units (RFU/h) in the presence of 18 nM target DNA and various concentrations of purified recombinant protein. (**B**) Strand transfer activity (in RFU/h) in the presence of 450 nM purified recombinant protein and the indicated concentration of target DNA. Lines are fits; error bars indicate ± s.e.m.

Our previous work showed that the primary resistance mutation R263K decreased integrase activity in cell-free assays [17]. Now, experiments with purified recombinant wild-type (wt) and mutated integrase proteins, i.e. IN_WT_, IN_H51Y_, IN_R263K_, and IN_H51Y/R263K_ at varying concentrations (Figure 1A and B) showed that IN_WT_ and IN_H51Y_ had comparable maximal strand transfer activity (100±3.7% and 107.7±10.7%, respectively) whereas IN_H51Y/R263K_ maximal activity was severely diminished (20.01±2.9%). Similar observations were made in the presence of variable concentrations of DNA substrate (Figure 1C). The sequential addition of R263K and the H51Y/R263K mutations resulted in an incremental loss in enzyme activity, while the effect of H51Y alone was innocuous.

### The addition of H51Y to R263K decreases HIV replication capacity

**Figure 2:**
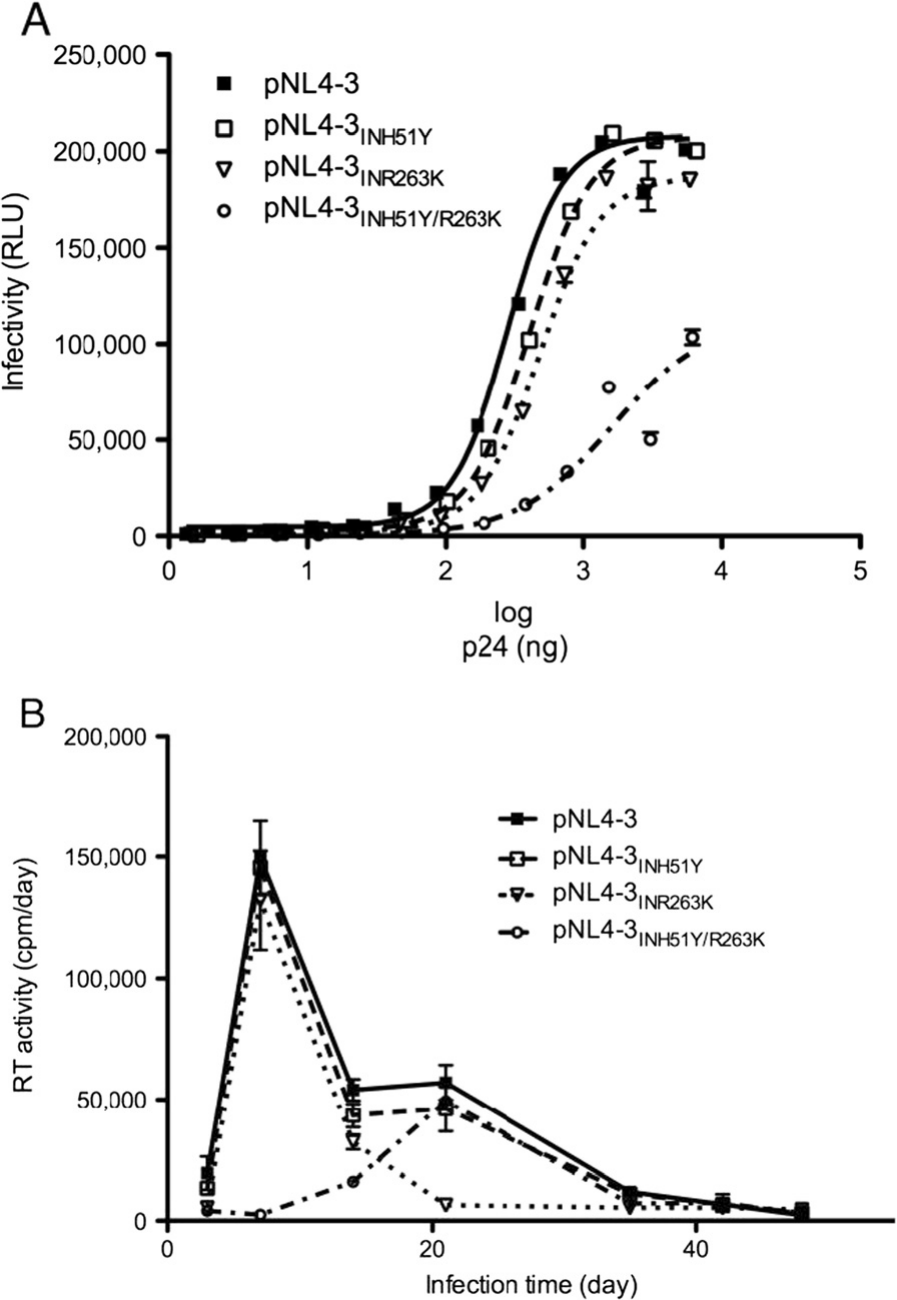
**Effects of the H51Y and R263K mutations on HIV infectivity and replicative fitness.** (**A**) pNL4.3_IN(WT)_, pNL4.3_IN(H51Y)_, pNL4.3_IN(R263K)_, and pNL4.3_IN(H51Y/R263K)_ infectivity were measured by quantifying luciferase activity in relative luminescent units (RLU) produced by TZM-bl cells infected with increasing concentrations of virus (in ng of p24 antigen). Lines are fits. (**B**) Reverse transcriptase (RT) activity was measured as counts per minute (cpm) per day in the culture fluids of PM1 cells infected with pNL4.3_IN(WT)_, pNL4.3_IN(H51Y)_, pNL4.3_IN(R263K)_, or pNL4.3_IN(H51Y/R263K)_ virus. Error bars indicate ± s.e.m.

To determine whether this biochemical defect also applied to replication capacity in cell culture, we assessed viruses that were either wt or contained these various mutations, i.e. pNL4.3_IN(WT)_, pNL4.3_IN(H51Y)_, pNL4.3_IN(R263K)_, and pNL4.3_IN(H51Y/R263K)_ (Figure 2). Although the single mutations modestly diminished HIV replication, the H51Y/R263K combination resulted in a dramatic impairment of viral infectivity (Figure 2A) and viral fitness (Figure 2B). The negative effect of the H51Y/R263K combination on HIV replication capacity was also observed in the PhenoSense® integrase replication assay (Table 2). Together with our biochemical results, these data suggest that the H51Y mutation does not compensate for the primary resistance mutation R263K [17] but rather has an additional detrimental effect on viral fitness when R263K is present.

### The addition of H51Y to R263K decreases HIV integration

**Figure 3:**
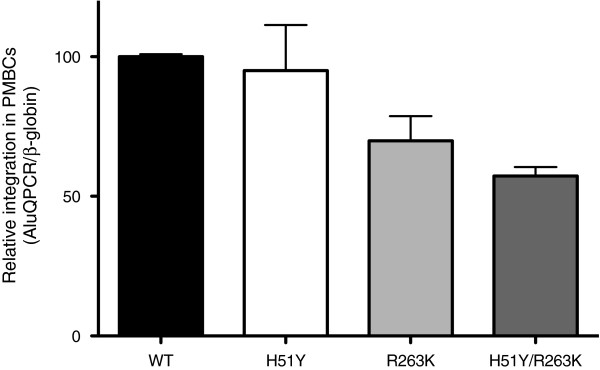
**Effects of the H51Y and R263K mutations on HIV integration.** Integrated HIV DNA was quantified by qPCR in primary human PBMCs infected with wild-type virus and with viruses containing the H51Y, R263K, and H51Y/R263K mutations for 72h. DNA was normalized for β-globin gene content relative to the the signal detected for wild-type virus, arbitrarily set at 100%. Error bars indicate ± s.e.m.

To determine whether the observed defect in HIV replication capacity conferred by the R263K and H51Y mutations was due to a decrease of HIV DNA integration, we used an Alu-mediated qPCR assay to monitor integration in primary human peripheral blood mononuclear cells (PBMCs) infected with pNL4.3_IN(WT)_, pNL4.3_IN(H51Y)_, pNL4.3_IN(R263K)_, and pNL4.3_IN(H51Y/R263K)_ (Figure 3). In agreement with our biochemical results, we found that the H51Y mutation had no effect on HIV integration. In contrast, the R263K mutation and the H51Y/R263K combination were associated with decreased integration, similar to the reduced enzymatic activity measured with the purified mutated recombinant integrase proteins.

### *In silico* studies of H51Y mutant integrase

**Figure 4:**
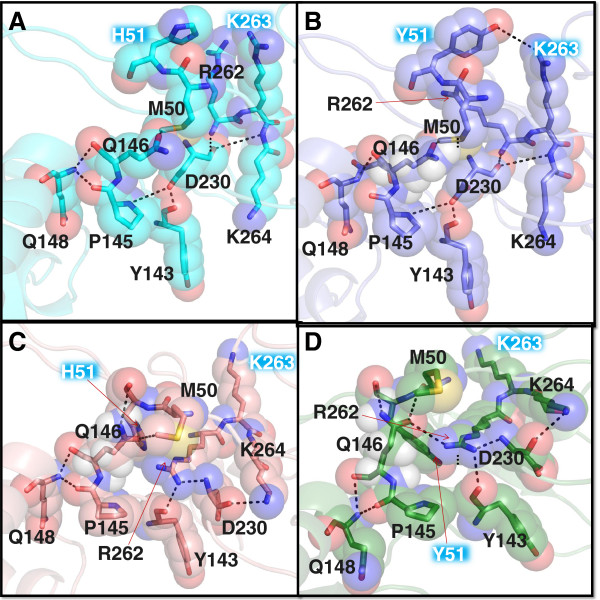
**Effect of the H51Y and R263K mutations on integrase structure.** Effect of residues at position 51 and 263 on local side-chain electrostatic interactions and side-chain mobility of: **A**. IN_wt_ (turquoise backbone); **B**. IN_H51Y_ (purple backbone); **C**. IN_R263K_ (salmon backbone) and **D**. IN_H51Y/R263K_ (Dark green backbone). Highlighted residues are shown as sticks within partly transparent space-filling structures coloured according to standard atomic colouration. Suspected hydrogen-bonding (<3.5Å) and electrostatic interactions (<4.5 Å) are represented by dotted black lines.

To gain insight into the effect of the H51Y and R263K mutations on susceptibility to DTG, we performed structural modeling of HIV integrase in the presence of each mutation alone and in combination. A comparison of wild-type IN (Figure 4A) to H51Y IN (Figure 4B) revealed no significant differences in secondary structure; however, a comparison of wild-type to R263K (Figure 4C) and H51Y/R263K (Figure 4D) demonstrated incremental disruptions in orientation of R262 and K264 that may contribute to viral DNA interactions [22,23], resulting in a larger scale disruption of electrostatic interactions in the C-terminus of integrase, which are transferred to key residues involved in INSTI drug resistance, i.e. P145, Q148, and Y143 [23]. Additionally, in the case of both R263K and H51Y/R263K, the orientation of the residue at position 51 is inverted (Figure 4C and 4D), which may have an impact on HIV-1 DNA binding ability, explaining the loss in fitness of the R263K and H51Y/R263K viruses. Docking of DTG to the model active sites showed favorable binding in all active sites, albeit with reduced apparent affinity in the R263K and H51Y/R263K models.

## Discussion

*In vitro* selection experiments with both DTG and another second-generation compound termed MK-2048 have identified several mutations within the integrase coding region, including G118R, S153Y, and R263K that confer low-level resistance to these drugs [17,24-27]. Additionally, during selection studies in primary human cord blood mononuclear cells, it appeared as though a secondary mutation H51Y was often present [17]. Here, we have shown that the combination of mutations at positions H51Y and R263K define a unique resistance pathway for DTG. Typically, the emergence of a first resistance mutation usually results in a decrease in relevant enzymatic activity, as has been observed for multiple drugs in different drug classes, whereas secondary mutations are often compensatory and partially or totally restore both enzymatic function and viral fitness [28,29]. In contrast, we demonstrate that the combination of R263K together with H51Y simultaneously increased levels of resistance against DTG while diminishing both viral replicative capacity and integrase strand transfer activity. Perhaps, more importantly, this combination of mutations further diminished the ability of viral DNA to integrate within the host cell genome beyond the deficit associated with the R263K mutation alone. The H51Y substitution on its own did not affect either DTG drug resistance, viral replication capacity, or integrase strand transfer enzymatic activity, as tested both biochemically and in cell-based assays. We believe that the fitness cost of the H51Y/R263K combination may explain the absence of resistance mutations in integrase inhibitor-naïve patients treated to date with DTG [6,12,15,16], since viruses that possess these two mutations in tandem may replicate so poorly as to be undetectable by the conventional assays that were employed for detection of drug resistance in the above-referenced clinical studies.

A more important issue may be the potential value of using a drug to select for mutations that severely compromise both viral replication capacity and the ability of viral DNA to integrate into host cells. In addition, we have continued our DTG tissue culture selection efforts for over one year and have not yet identified any potential compensatory mutations that might restore integrase function while increasing levels of drug resistance above those seen with the combination of R263K and H51Y. This may be related to either the low replication capacity of viruses containing the H51Y/R263K combination or the poor integrase strand transfer capacity of enzymes containing these substitutions, or both. The findings presented here may be related to the fact that DTG posesses a very long residency time on the HIV integrase enzyme [30]. Of course, it is possible as well that tissue culture drug selection protocols are inadequate to select for all mutations of relevance.

We agree that definitive results that further define drug resistance to DTG, including additional DTG-related mutations, may arise in first-line treatment, especially from the clinical use of DTG after its approval in settings that may not always emphasize the importance of adherence to antiretroviral treatment regimens in the same way as do registrational clinical trials. However, if the results reported here are further clinically validated, consideration should be given to exploring the possibility that the H51Y/R263K combination of mutations, perhaps in concert with other strategies, might reduce or prevent new cycles of HIV DNA integration into host cells.

We are currently in the process of studying whether the R263K and H51Y mutations in simian immunodeficiency virus (SIV) have similar effects as those described here. If so, this would serve to justify further studies in macaque monkeys that employ our mutated viruses, both in the presence and absence of DTG, aimed at achieving retroviral eradication from infected hosts. The use of mutated HIV in humanized mouse models could also be tested.

One caveat of the above arguments is that viruses that fail to achieve integration will not help us to deal with the problem of the HIV reservoir in individuals previously infected by HIV, even if such subjects have been treated with drugs such as DTG. However, it is possible that the withholding of all anti-HIV drugs from treated subjects might result in an activation of latent viruses from reservoirs. In this context, viruses that fail to achieve efficient integration, such as those decribed here, might fail to effectively repopulate reservoirs and multiple cycles of on/off therapy might diminish the size of the latent reservoir over time. While awaiting the results of further clinical studies involving DTG, these are concepts that could be studied in animal models.

## Conclusions

Our findings suggest that DTG may be intrinsically resistant to the emergence of resistance and that this drug should be used in first-line therapy to minimize the emergence of possible drug resistance. The finding that a secondary mutation, i.e. H51Y, may simultaneously reduce viral replication and enzymatic activity, while augmenting levels of drug resistance in the presence of a primary resistance mutation, i.e. R263K, could potentially be advantageous not only for HIV treatment but for strategies aimed at HIV eradication as well.

## Methods

### Cells and antiviral compounds

TZM-bl, 293T, and PM1 cells were used as described [17]. Human primary peripheral blood mononuclear cells (PBMCs) were isolated from whole blood of healthy donors using Ficoll-Hypaque and stimulated with 10 μg/ml phytohemagglutinin A (PHA) and 20 U/ml human interleukin-2 (IL-2) for 72 h. Merck & Co., Inc. and ViiV Healthcare Ltd. kindly provided raltegravir (RAL) and dolutegravir (DTG), respectively. Efavirenz (EFV) was obtained from the NIH AIDS Research and Reference Reagent Program.

### Integrase strand-transfer activity assay

Integrase strand transfer reactions with recombinant purified proteins were carried out as published [17], with the major difference being the use of pre-processed LTR DNA (sense amino-group-5^′^-ACCCTTTTAGTCAGTGTGGAAAATCTCTAGCA-3^′^ and antisense 5^′^-ACTGCTAGAGATTTTCCACACTGACTAAAAG-3^′^). LTR DNA was covalently linked to Costar DNA Bind 96-well plates (Corning) and the plates were blocked and washed as described [17]. Purified integrase proteins were incubated for 30 minutes before the biotinylated target DNA (sense 5^′^-TGACCAAGGGCTAATTCACT-3Bio and antisense 5^′^-AGTGAATTAGCCCTTGGTCA-3Bio) was added, followed by an additional incubation of 1 h at 37°C for the strand-transfer reaction to occur. After washes, strand-transfer was quantified through the use of Eu-labelled streptavidin (Perkin Elmer), as described previously [17].

### Generation of replication-competent genetically homogenous HIV-1

pNL4.3_IN(R263K)_ has been described previously [17]. To study the H51Y mutation and the H51Y/R263K combination, pNL4.3_IN(H51Y)_ and pNL4.3_IN(H51Y/R263K)_ were generated by site-directed mutagenesis using H51Y primers (sense: 5^′^-CTAAAAGGGGAAGCCATGTATGGACAAGTAGACTGTA-3^′^ and antisense: 5^′^-TACAGTCTACTTGTCCATACATGGCTTCCCCTTTTAG-3^′^), and the QuickChange II XL Site-Directed mutagenesis kit (Stratagene). The presence of the mutations was confirmed by sequencing. Genetically homogenous viruses were produced by transfecting wild-type and mutated pNL4.3 plasmids into 293T cells as described previously [17].

### HIV susceptibility to antiretroviral compounds

HIV susceptibilities to DTG, RAL, and EFV were measured in TZM-bl cell at 48 h after infection as described previously [17]. Fifty percent inhibitory concentrations (IC_50_s) and 95% confidence intervals were calculated on the basis of at least three experiments by using GraphPad Prism 4.0 Software.

### Monogram biosciences PhenoSense replication capacity and phenotyping assays

HIV replication capacity and susceptibilities to DTG, RAL, and EFV were measured as previously described [31]. Briefly, murine leukemia virus envelope-pseudotyped viruses bearing the integrase H51Y and R263K mutations and a luciferase reporter gene were used to inoculate human embryonic kidney HEK-293 cells. The resultant luciferase activity was used to calculate changes in HIV replication capacity relative to a wild-type reference strain. Drug susceptibility was expressed as a fold-change in IC_50_.

### HIV infectivity and replication capacity

HIV infectivity was evaluated using a noncompetitive short-term infectivitiy assay in TZM-bl cells as previously described [17]. HIV replication capacity was measured over time in PM1 cells by quantifiying RT activity (in counts per minute) in culture fluids as described previously [32].

### Determination of HIV integration in PBMCs

A quantitative PCR (qPCR) assay for integrated DNA in primary human PBMCs was performed as previously described [17,24]. Briefly, cellular DNA was extracted using the DNeasy blood and tissue extraction kit from Qiagen and amplified in a two-step PCR. The second step was performed with Platinum qPCR SuperMix-UDG (Invitrogen) on a Corbett Rotor-Gene 6000 (Corbett) by using the following conditions: 50°C for 2 min, 95°C for 2 min, and 50 repeats of 95°C for 10 s, 60°C for 20 s, and 72°C for 45 s. The samples were normalized for their β-globin gene content. Primers and probes have been described previously [24].

### *In silico* studies of HIV integrase

Molecular modeling of HIV integrase and DTG docking were performed with the PFV lead template structures PDBID: 3S3M [25] and PDBID: 3L2S [33] as previously described [17].

## Competing interests

The authors declare no competing financial interests.

## Authors’ contributions

TM designed and performed experiments, analysed data and wrote the manuscript; PKQ performed the molecular modeling analyses; N.O. performed experiments and analysed data; YH developed analytical tools; DNS performed experiments;YL, CJP and WH performed the PhenoSense® assays; and MAW supervised the project and revised the manuscript. All authors read and approved the final manuscript.
